# MicroRNA-1911-3p targets mEAK-7 to suppress mTOR signaling in human lung cancer cells

**DOI:** 10.1016/j.heliyon.2020.e05734

**Published:** 2020-12-19

**Authors:** Daniela Baccelli Mendonça, Joe Truong Nguyen, Fatima Haidar, Alexandra Lucienne Fox, Connor Ray, Halimah Amatullah, Fei Liu, Jin Koo Kim, Paul H. Krebsbach

**Affiliations:** a Department of Biologic and Materials Sciences, University of Michigan, Ann Arbor, MI, 48105, USA; b Biointerfaces Institute, University of Michigan, Ann Arbor, MI, 48105, USA; c Section of Periodontics, University of California, Los Angeles, School of Dentistry, Los Angeles, CA, 90095, USA

**Keywords:** MicroRNA-1911-3p, mEAK-7, mTOR signaling, Lung cancer, Cell proliferation, Cell migration, Cell biology, Gene expression, Gene regulation, Cancer research, Oncology

## Abstract

Regulation of mTOR signaling depends on an intricate interplay of post-translational protein modification. Recently, mEAK-7 (mTOR associated protein, eak-7 homolog) was identified as a positive activator of mTOR signaling via an alternative mTOR complex. However, the upstream regulation of mEAK-7 in human cells is not known. Because microRNAs are capable of modulating protein translation of RNA in eukaryotes, we conducted a bioinformatic search for relevant mEAK-7 targeting microRNAs using the Exiqon miRSearch V3.0 algorithm. Based on the score obtained through miRSearch V3.0, the top predicted miRNA (miR-1911-3p) was studied. miR-1911-3p mimics decreased protein levels of both mEAK-7 and mTORC1 downstream effectors p-S6 and p-4E-BP1 in non-small cell lung carcinoma (NSCLC) cell lines H1975 and H1299. miR-1911-3p levels and *MEAK7* mRNA/mEAK-7/mTOR signaling levels were negatively correlated between normal lung and NSCLC cells. miR-1911-3p directly interacted with *MEAK7* mRNA at the 3′-UTR to negatively regulate mEAK-7 and significantly decreased mTOR localization to the lysosome. Furthermore, miR-1911-3p significantly decreased cell proliferation and migration in both H1975 and H1299 cells. Thus, miR-1911-3p functions as a suppressor of mTOR signaling through the regulation of *MEAK7* mRNA translation in human cancer cells.

## 1 Introduction

Mammalian EAK-7, or MTOR associated protein, eak-7 homolog (mEAK-7), is a positive regulator of mTOR signaling that functions through the S6K2/4E-BP1 axis in human cells [1]. mEAK-7 is expressed predominantly in metastatic human cancer and forms a novel mTOR complex involving DNA-PK to promote S6K2 signaling and suppress 4E-BP1 [2]. It is well known that aberrant mTOR signaling results in diverse diseases such as diabetes type II, neurological disorders, and cancer [3], and thus a more complete understanding of the interactions of mTOR and mEAK-7 may be important to preventing or treating these human conditions.

mTOR is a member of the phosphatidylinositol-3 kinase-related kinase (PIKK) family [4]. These PIKKs typically have redundant functions and include ataxia-telangiectasia mutated (ATM), ataxia- and Rad3-related (ATR), and DNA-dependent protein kinase catalytic subunit (DNA-PKcs) [5]. PIKKs share redundancies to effectively regulate cell metabolism, DNA repair pathways, and genome surveillance. mTOR signaling is defined by two multi-protein complexes. mTOR complex 1 (mTORC1) targets S6K1/4E-BP1 and mTOR complex 2 (mTORC2) targets Akt [3]. Both complexes function in the lysosome, an essential cellular compartment for mTOR signaling [6, 7]. mEAK-7 is anchored at the lysosomal membrane and forms an alternative mTOR complex with DNA-PK to regulate mTOR signaling [1, 2]. We have posited that this novel complex is a third member of known mTOR complexes, mTORC3. Other research groups have identified an astrocyte-specific mTOR complex [8] and a rapamycin insensitive mTOR complex in human cancer cells [9]. These findings demonstrate the existence of multiple mTOR complexes and suggest the possibility that additional cell-type or disease-specific mTOR complexes may exist.

MicroRNAs (miRNAs) are short, non-coding RNA molecules that mediate gene silencing by guiding an intricate series of protein complexes to target specific motifs in the 3′ untranslated region (UTR) of messenger RNAs (mRNAs) [10]. The first miRNA, named lin-4, was reported in *Caenorhabditis elegans*, and was shown to be required for post-transcriptional repression of lin14, a protein that regulates the timing of several developmental processes in worms [11]. It was later discovered that this class of mRNA regulation was essential for eukaryotic development and was discovered in many different animal phyla [12]. Collectively across eukaryotes, these conserved RNA molecules are known as miRNAs [13]. Recently, it has been appreciated that mTOR signaling may also be modulated by post-transcriptional regulation through miRNA targeting mTOR and upstream regulators of mTOR [14]. Within diverse cancer types, miRNA expression profiles are substantially different compared to their normal tissue counterparts, and miR-21 overexpression directly activates aberrant mTOR signaling in many solid cancers [14, 15].

In this study, we hypothesized that miRNAs regulate mEAK-7 to modify mTOR signaling in cancer cells. We investigated the extent to which miR-1911-3p regulates mTOR signaling through mEAK-7.

## 2 Materials and methods

### 2.1 Cell culture

Human non-small lung cancer cell lines H1975 (ATCC CRL-5908) and H1299 (ATCC CRL-5803) were obtained from American Type Tissue Collection (ATCC) and maintained in Dulbecco's Modified Eagle Medium (DMEM) (Thermo Fisher Scientific (TFS), cat# 11995-073) containing 10% fetal bovine serum (FBS) (TFS, cat#10437-036). A human bronchial epithelial cell line BEAS-2B (ATCC CRL-9609) was obtained from ATCC and maintained in Bronchial Epithelial Cell Growth Basal Medium (Lonza, CC-3171) containing growth factors and supplements from BEGM BulletKit (Lonza, CC-4175).

### 2.2 miRNA or plasmid transfection

miRNA negative control (TFS, cat# 4464059), miR-1911-3p mimic (TFS, cat# 4464067), and miR-1911-3p inhibitor (TFS, cat# 4464085) were obtained from TFS. pmirGLO Dual-Luciferase miRNA Target Expression Vector was purchased from Promega (cat# E1330). On the day prior to transfection, 500,000 cells were seeded into 60-mm tissue culture plates. Then, cells were transiently transfected with 100 nM of miRNAs for 48 h, using Lipofectamine RNAiMAX Transfection Reagent (TFS, cat# 13778-150). For plasmid transfection, we used FUGENE 6 Transfection reagent (Promega, cat# E2691). For miRNA and DNA co-transfection experiments, both transfection reagents were added.

### 2.3 Cell proliferation and migration

Cells were seeded at a density of 500,000 into 60-mm tissue culture plates and grown for 24 h. Cells were transfected with 100nM of miR-control or miR-1911-3p mimic. For cell proliferation, 48 h after transfection, 200,000 cells were seeded into 100-mm tissue culture plates and counted after days 3 and 5 using the LUNA Automated Cell Counter (Logos Biosystems (LB), cat# L10001) along with LUNA Cell Counting Slides (LB, cat# L12003) and AO-PI dye (LB: cat# F23001). For cell migration, 48 h after transfection, cells were seeded at 50,000 cells per well into CIM 16-well plates (ACEA Biosciences, cat# 05665817001). Cell migration was captured in real time for 48 h using the xCELLigence System, RTCA DP instrument (ACEA Biosciences, cat# 00380601050) and processed by RTCA Software 2.0.

### 2.4 Microarray and quantitative real-time PCR (qRT-PCR)

Total RNA was isolated using miRNeasy Mini kit (Qiagen, cat# 217004), according to manufacturer's instructions. Microarray analysis was performed by the Advanced Genomics Core (University of Michigan). The quality of each RNA sample was analyzed using a 2100 Bioanalyzer (Agilent Technologies). Biotinylated cDNA were prepared from 400 ng of total RNA using the GeneChip Whole Transcript PLUS Reagent Kit (Applied Biosystems) according to the Affymetrix Plus Whole Transcript kit protocol (Manual P/N 703174 Rev. 2). Following labeling procedure, 2.76 ug of cDNA were hybridized at 48 °C on Human Gene ST 2.1 Array Strips, and washed, and stained using the Affymetrix Gene Atlas system (software version 2.0.0.460). Subsequently, arrays were scanned using the Affymetrix Gene Atlas system (software version 1.0.4.267). For data processing, the Robust Multi-Array Average (RMA) method was used to fit log2 expression values to the data using the oligo bioconductor package in R version 3.3.0. The bioinformatics analysis of microarray data was performed by iPathwayGuide software of Advaita Corporation (www.Advaitabio.com). For qRT-PCR, 1 μg of RNA was reverse transcribed using SuperScript VILO cDNA synthesis kit (TFS, cat# 11754050), and cDNA was used for qRT-PCR using mEAK-7 (TLDC1) primer (TFS, Hs00297285_m1, cat# 4331182) in an Applied Biosystems 7900HT Real Time PCR System (TFS). mRNA levels were normalized to ACTB or GAPDH as a housekeeping gene. For microRNA analysis, microRNA was isolated using a Mirpremier microRNA isolation kit (Sigma, cat# SNC50-1KT), according to manufacturer's instructions. MicroRNAs (100 ng) were first polyadenylated and then reverse transcribed using MystiCq microRNA cDNA Synthesis Mix kit (Sigma, cat# MIRRT). cDNA was used for qRT-PCR using miR-1911-3p primer (GeneCopoeia, cat# HmiRQP0266) and MystiCq microRNA SYBR Green qPCR ReadyMix (Sigma, cat# MIRRM00) in a CFX96 Real Time PCR System (Bio-Rad). microRNA levels were normalized to RNU6-1 (MystiCq microRNA qPCR control primer, Sigma, cat# MIRCP00001).

### 2.5 Western blot

Cells were harvested and lysed with cold NP-40 lysis buffer (50 mM tris, 150 mM NaCl, and 1.0% NP-40 at pH 8.0). 50 μg of protein lysate was separated in Novex WedgeWell 4–20% gradient tris-glycine gels (TFS, cat# XP04205BOX), and transferred to PVDF membranes. Membranes were blocked with 5% non-fat dry milk in 1x TBST, incubated with primary antibodies overnight at 4 °C, and then with HRP-conjugated secondary antibodies for 1 h at room temperature. Blots were developed using SuperSignal West Pico (TFS, cat# 34078) or Femto (TFS, cat#34095) Chemiluminescent Substrate Solution. The primary antibodies used were: mouse monoclonal antibody against mEAK-7 (KIAA1609), which was obtained from Origene Technologies (clone OTI12B1, formerly 12B1, cat# TA501037). All antibodies from Cell Signaling Technology were: GAPDH (cat# 2118S), β-actin (4970), (Ser235/236) p-S6 (2211), (Ser240/244) p-S6 (2215), S6 ribosomal protein (2217), (Thr37/46) p–4E-BP1 (9459), (Ser65) p–4E-BP1 (9451), (Thr70) p–4E-BP1 (13396), and 4E-BP1 (9452). Antibodies p-S6, S6, and 4E-BP1 were used at 1:3000 dilution and the remainder at 1:1000 dilution in 5% bovine serum albumin (BSA) in TBST buffer with 0.04% sodium azide. Secondary antibodies for immunoblot analysis: 1:4000 dilution for an α-mouse IgG horseradish peroxidase (HRP) conjugate (Promega, cat# W4021) and 1:7500 dilution for an α-rabbit IgG HRP conjugate (Promega, cat# W4011).

### 2.6 Cloning of 3′-UTR of MEAK7 and miRNA analysis via luciferase assay

Oligonucleotides containing the 3′-UTR of *MEAK7* mRNA with the potential miR-1911-3p binding site, were cloned into Dual-Luciferase miRNA Target Expression Vector pmirGLO (Promega). The resulting ligated product (pmirGLO-3′-UTR-*MEAK7*) was transformed into DH5α competent cells (TFS, cat# EC0112). Plasmid DNA was confirmed by DNA sequencing (University of Michigan DNA Sequencing Core). Cell were co-transfected with 100 nM of miRNA-control or miRNA-1911-3p mimic in combination with 0.5μg of pmirGLO or pmirGLO-3′-UTR-*MEAK7*. After 48 h, cells were lysed in passive lysis buffer. Dual Luciferase Assay (Promega, cat# E1910) was performed according to the manufacturer's instructions. Luciferase activities were normalized with basal Renilla luciferase activities. The empty pmirGLO vector (pmirGLO Control) was set to 100% for luciferase activity.

### 2.7 Cell immunofluorescence analysis

Cells transfected with control miRNA or 1911-3p miRNA were seeded (62,500 cells/cm^2^) into a two-well Nunc Lab-Tek II Chamber Slide System (TFS, catalog #12-565-5) for 24 h. Then, cells were fixed with Z-Fix solution (Anatech LTD, catalog #170) for 10 min at room temperature, washed three times in PBS, and incubated with the following: unmasking solution (PBS, 2N HCl, and 0.5% Triton X-100) for 10 min, quenching solution (TBS and 0.1% sodium borohydride) for 10 min, permeabilization solution (PBS and 0.02% Triton X-100) for 10 min, and 5% BSA for 1 h. Cells were incubated overnight at 4 °C with primary antibodies. Next, slides were washed with PBS and incubated in secondary antibodies for 1 h at room temperature. A Nikon Ti2 Eclipse confocal microscope (×60 oil magnification) was used to capture images. Images were captured with 1/32 frames/s, 1024 × 1024 image capture, 1.2 airy units, 0x line averaging, appropriate voltage, and power settings optimized per antibody. No modification was done, except image sizing reduction for figure preparation. Identical threshold settings captured images across three to five individual fields (10–15 cells) per condition, with the data representing at least three independent experiments. For quantitative analyses, confocal images in six random fields per experimental condition were used for counting the number of cells showing mTOR/LAMP2 colocalization. Primary antibodies for immunofluorescence were as follows: LAMP2 (1:1500, SCB, catalog #sc-18822) and mTOR (1:1000, CST, catalog #2983S). Both antibodies were used with a working volume of 1.5 ml in 5% BSA in PBS. Secondary antibodies for immunofluorescence were as follows: Anti-mouse IgG (H + L), F (ab')2 Fragment (Alexa Fluor® 488 Conjugate) (CST, catalog #4408) and anti-rabbit IgG (H + L), F (ab')2 Fragment (Alexa Fluor® 594 Conjugate) (CST, catalog #8889). Both antibodies were used at a concentration of 1:1500 with a working volume of 1.5ml in 5% BSA in PBS. DAPI stain was used for DNA.

### 2.8 Statistical analysis

For statistical analysis, data are presented as mean ± S.E or S.D. Significance of the difference between two measurements was determined by unpaired Student's t-test. Paired Student's t-test was used for cell proliferation and migration. All experiments were repeated at least three times in all cell lines.

## 3 Results

### 3.1 miR-1911-3p decreases mEAK-7 protein levels to suppress mTOR signaling

**Figure 1:**
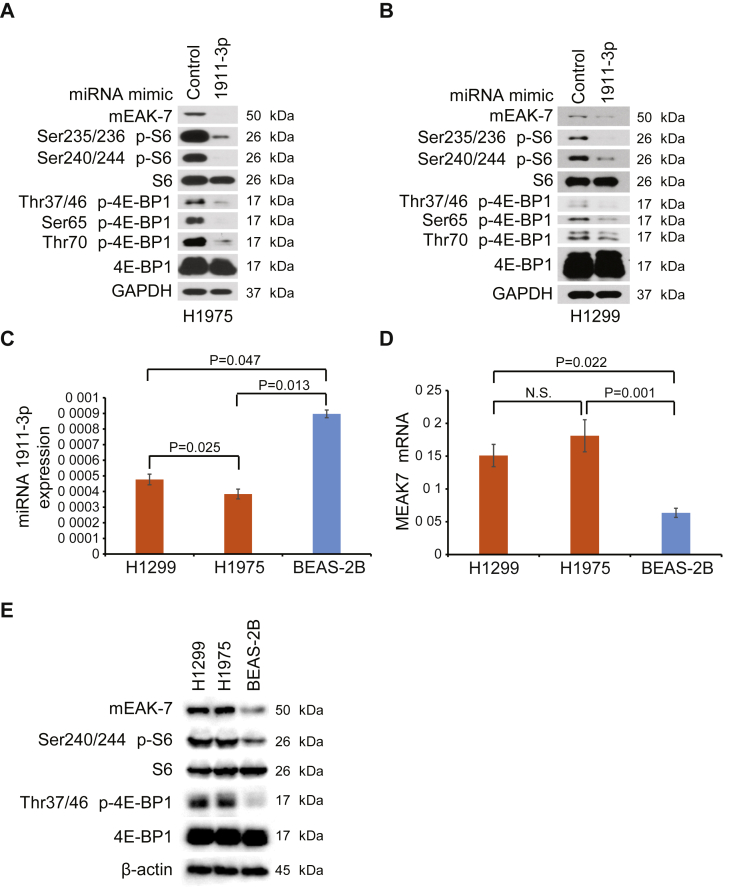
miR-1911-3p is an effector for mEAK-7 and mTOR signaling in human lung cancer cells. H1975 (A) and H1299 (B) cells were transfected with miR-control or miR-1911-3p mimic for 48 h to evaluate mEAK-7 and mTOR signaling. GAPDH was used as a loading control. See full images in Supplementary Figure S1. Expression of miR-1911-3p (C) and *MEAK7* (D) in H1299, H1975, and BEAS-2B cells was quantified by qPCR. microRNA and mRNA levels were normalized to RNU6-1 and GAPDH, respectively. Data are expressed as means ± SD of 3 independent experiments. n.s., not significant. (E) Expression of mEAK-7 and mTOR signaling in H1299, H1975, and BEAS-2B cells was analyzed by Western blotting. β-actin was used as a loading control. See full images in Supplementary Figure S2. All experiments were repeated at least three times.

Several cancer cell lines synthesize mEAK-7 protein [1]. Interestingly, while mEAK-7 protein is predominantly detected in human cancer cell lines, it is not consistently expressed in all cancers. To investigate the extent to which miRNAs modify mEAK-7 expression, the Exiqon database was assessed with the Exiqon miRSearch V3.0 algorithm (www.exiqon.com/miRSearch), and miR-1911-3p was identified as a top predicted target for mEAK-7 (Table 1). Non-small cell lung carcinoma (NSCLC) cells from ATCC, H1975 and H1299 were used for this study. To test the effect of this potential miRNA on mEAK-7 expression and mTOR signaling, H1975 and H1299 cells were treated with 100 nM mimics of this miRNA or miRNA controls for 48 h in DMEM medium containing 10% serum. miR-1911-3p mimics resulted in a significant decrease in mEAK-7 protein levels, thereby suppressing mTOR signaling through (Ser^235/236^) p-S6, (Ser^240/244^) p-S6, (Thr^37/46^) p-4E-BP1, (Ser^65^) p-4E-BP1, and (Thr^70^) p-4E-BP1 in H1975 (Figure 1A) and H1299 cells (Figure 1B). Thus, we have identified a novel miRNA that targets *MEAK7* to suppress mTOR signaling.

**Table 1:** Exiqon miRSearch for potential mEAK-7 (KIAA1609) related miRNAs.

#	microRNA	Accession number
1	has-miR-1911-3p	MIMAT0007886
2	has-miR-1233-5p	MIMAT0022943
3	has-miR-1294	MIMAT0005884
4	has-miR-149-3p	MIMAT0004609
5	has-miR-202-3p	MIMAT0002811
6	has-miR-3192-5p	MIMAT0015076
7	has-miR-3649	MIMAT0018069
8	has-miR-4314	MIMAT0016868
9	has-miR-4529-3p	MIMAT0019068
10	has-miR-4648	MIMAT0019710

To investigate the basal expression levels of miR-1911-3p and *MEAK7* in lung cancer and normal lung cells, miR-1911-3p and *MEAK7* expression was examined by quantitative RT-PCR in H1299, H1975, and human bronchial epithelial cell BEAS-2B. miR-1911-3p expression levels were low in both H1299 and H1975 cells, but higher in BEAS-2B cells (BEAS-2B:H1299, fold change: 1.88; BEAS-2B:H1975, fold change: 2.33) (Figure 1C). In contrast, *MEAK7* mRNA levels were higher in both H1299 and H1975 cells compared to BEAS-2B cells (H1299:BEAS-2B, fold change: 2.37; H1975:BEAS-2B, fold change: 2.85) (Figure 1D). Furthermore, the protein levels of mEAK-7 and mTOR signaling were significantly higher in both H1299 and H1975 cells compared to BEAS-2B cells (Figure 1E). Thus, these data showed a negative correlation between miR-1911-3p levels and *MEAK7*/mEAK-7/mTOR signaling levels in normal lung and NSCLC cells.

### 3.2 miR-1911-3p targets MEAK7 mRNA at 3′-UTR

MicroRNAs are a class of small, non-coding RNAs that have important regulatory roles in biological processes such as cell metabolism, proliferation, differentiation, migration, and apoptosis [16, 17]. miRNAs most often interact with the 3′-UTR of the target mRNA and either block translation into protein, or promote RNA degradation [16, 17]. miRNAs have also been associated with the onset and progression of human pathological conditions, including several types of cancer, exhibiting either oncogenic or suppressor functions, depending on the genes they regulate and the cellular context. A linkage between miRNAs and NSCLC progression has been described and specific miRNAs promote tumor metastasis [18, 19, 20, 21]. Interestingly, a single miRNA can regulate more than one target gene and this is particularly important in cancer because it is a heterogenic disease and typically cannot be treated by targeting a single gene [22]. Cancer related miRNAs and their target genes have important clinical applications as well [22].

**Figure 2:**
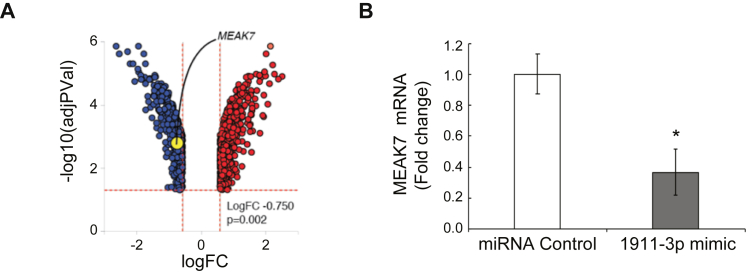
miR-1911-3p decreases *MEAK7* mRNA levels. (A) H1975 cells were treated with miR-control or miR-1911-3p mimic for 48 h. For Microarray analysis, RNA was isolated and samples were processed and hybridized to the Affymetrix Human Gene 2.1 ST Array. Microarray analysis was performed using Advaita Bioinformatics. *MEAK7* is highlighted in yellow in The Volcano plot. (B) *MEAK7* mRNA levels were determined by qRT-PCR. Actin Beta (ACTB) was used as loading control for qRT-PCR experiments. All experiments were repeated at least three times. Data are expressed as means ± SE of 3 independent experiments (∗*P* < 0.05).

**Figure 3:**
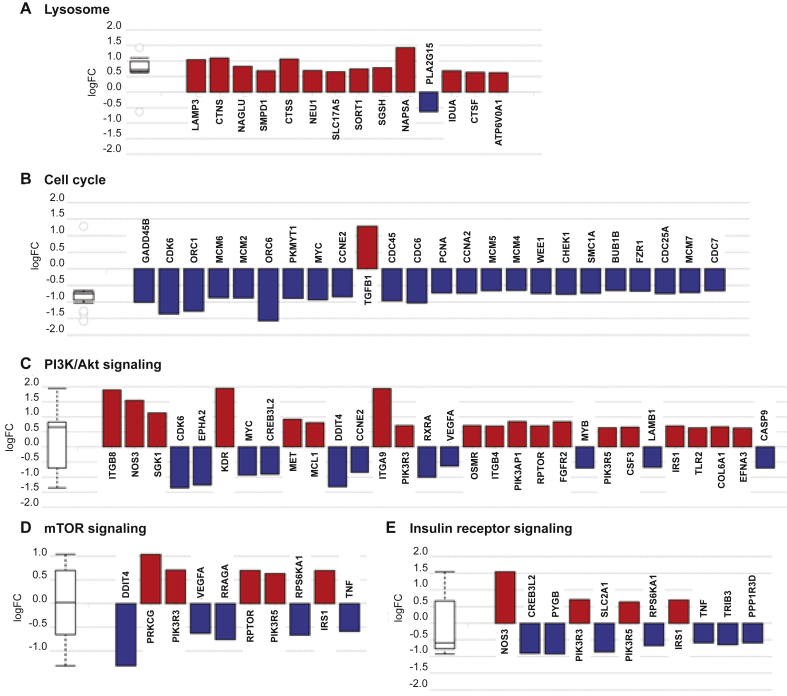
Microarray data for genes regulated by miR-1911-3p. H1975 cells were treated with miR-control or miR-1911-3p mimic for 48 h. RNA was isolated and samples were processed and hybridized to the Affymetrix Human Gene 2.1 ST Array. Microarray analysis was performed using Advaita Bioinformatics. (A) Bar graph of lysosomal genes. (B) Bar graph of cell cycle genes. (C) Bar graph of PI3K/Akt signaling genes. (D) Bar graph of mTOR signaling genes. (E) Bar graph of insulin receptor signaling genes.

To test whether miR-1911-3p regulates *MEAK7* mRNA and other target genes in NSCLC cells, H1975 cells were transfected with miR-control or miR-1911-3p mimic for 48 h. Total RNA was isolated and microarray analysis was performed. A volcano plot of the array data demonstrated that 1,370 genes were significantly and differentially expressed in miR-1911-3p transfected cells compared to controls (Figure 2A) with the data represented in terms of their measured expression change (x-axis) and the significance of the change (y-axis). Microarray data demonstrated that *MEAK7* levels were down-regulated by more than 2-fold (Figure 2A). Quantitative real time PCR (qRT-PCR) data confirmed the microarray results, showing a decrease in *MEAK7* gene expression by more than 2-fold (Figure 2B). For other target genes, microarray analysis revealed the top deregulated genes by miR-1911-3p (Table 2). The most positively regulated gene was *EGR1*. EGR1 functions as a transcriptional regulator [23]. EGR1 induces the tumor cell apoptosis via upregulating tumor suppressors, NAG-1 and PTEN directly. Studies suggested that *EGR1* functions as a tumor suppressor gene [23]. The most negatively regulated gene was *PEG10*. PEG10 is highly expressed in a variety of cancers containing lung cancer compared to normal tissues and promotes cancer proliferation and metastasis [24]. These data indicated that miR-1911-3p regulates both a tumor suppressor gene (*EGR1*) and oncogene (*PEG10)* to inhibit tumorigenesis. In addition, microarray analysis determined the most impacted pathways by miR-1911-3p (Table 3). miR-1911-3p significantly regulated the genes involving in mTOR signaling related pathways (cell cycle, PI3K-Akt signaling, and lysosome) and mTOR signaling pathway. In the deregulated mTOR signaling genes, seven of nineteen genes (MIOS, SLC3A2, ATP6V1C2, SLC7A5, RRAGA, LAMTOR3, and FNIP2) were genes related to lysosomal regulation of mTOR by amino acids (Table 4), suggesting that miR-1911-3p may target other mTOR signaling related genes as well as *MEAK7* directly or indirectly. Interestingly, microarray data suggested that miR-1911-3p mimic selectively increased lysosome-related gene expression (Figure 3A) and selectively decreased cell cycle-related gene expression (Figure 3B) compared to other signaling-related gene expression (Figure 3, C-E).

**Table 2:** The top deregulated genes obtained from the microarray analysis.

Ranking	Gene symbol	LogFC	p-value
1	EGR1	2.147	1.36E-06
2	PEG10	-2.641	1.36E-06
3	TOMM5	-1.95	1.36E-06
4	TMEM158	-2.287	2.01E-06
5	NA	-2.545	2.36E-06
6	PUDP	-1.783	2.36E-06
7	CLDN1	2.011	2.36E-06
8	CYR61	-1.733	4.88E-06
9	STC1	-2.209	4.88E-06
10	SHCBP1	-2.118	4.88E-06

**Table 3:** The most impacted pathways obtained from the microarray analysis.

Ranking	Pathway name	# genes (Deregulated∖All)	p-value
1	Influenza A	26∖77	5.43E-06
2	Hepatitis C	23∖71	6.68E-06
3	Cell cycle∗	24∖69	7.34E-06
4	MAPK signaling	35∖138	1.05E-05
5	IL-17 signaling	18∖47	2.17E-05
6	MicroRNA in cancer	30∖142	2.73E-05
7	Epstein-Barr virus infection	27**∖**94	5.60E-05
8	DNA replication	11∖26	7.27E-05
9	Ferroptosis	8∖16	1.76E-04
10	Metabolic	99**∖**603	1.87E-04
11	mTOR signaling∗	19**∖**68	1.98E-04
16	PI3K-Akt signaling∗	34∖147	3.90E-04
21	Lysosome∗	14∖51	0.002

∗ mTOR signaling related pathway.

**Table 4:** The deregulated mTOR signaling genes obtained from the microarray analysis.

Ranking	Gene symbol	LogFC	p-value
1	SGK1	1.128	3.45E-05
2	DDIT4	-1.321	1.41E-04
3	WNT7B	-1.288	1.58E-04
4	MIOS∗	-1.013	1.64E-04
5	PRKCG	1.038	1.70E-04
6	SLC3A2∗	-0.9	2.03E-04
7	PIK3R3	0.708	2.67E-04
8	FGD5	0.885	3.15E-04
9	ATP6V1C2∗	0.885	3.91E-04
10	SLC7A5∗	-0.657	5.84E-04
11	LRP5	0.649	7.80E-04
12	RRAGA∗	-0.765	8.91E-04
13	RPTOR	0.702	0.001
14	WNT9A	-0.8	0.002
15	RPS6KA1	-0.671	0.003
16	IRS1	0.698	0.004
17	TNF	-0.589	0.005
18	LAMTOR3∗	-0.692	0.014
19	FNIP2∗	0.594	0.026

∗ The genes related to lysosomal regulation of mTOR by amino acids.

**Figure 4:**
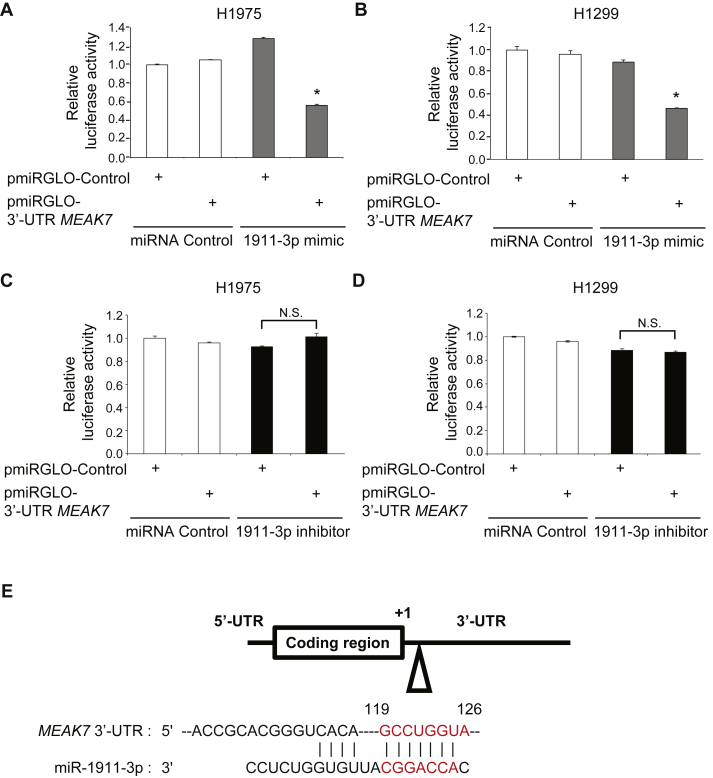
miR-1911-3p targets *MEAK7* mRNA at 3′-UTR. H1975 (A) and H1299 (B) cells were transiently co-transfected with 100 nM of miR-control or miR-1911-3p mimic in combination with 0.5μg of the empty luciferase reporter (pmirGLO) or pmirGLO-3′-UTR *MEAK7*. Luciferase activities were measured and normalized with basal Renilla luciferase activities. H1975 (C) and H1299 (D) cells were transiently co-transfected with 100 nM of miR-control or miR-1911-3p inhibitor in combination with 0.5μg of the empty luciferase reporter (pmirGLO) or pmirGLO-3′-UTR *MEAK7*. Luciferase activities were measured and normalized with basal Renilla luciferase activities. (E) Predicted consequential pairing of *MEAK7* 3′-UTR and miR-1911-3p. Pairing was performed using TargetScan (www.targetscan.org). All experiments were repeated at least three times. Data are expressed as means ± SE of 3 independent experiments (∗*P* < 0.05). n.s., not significant.

With evidence that miR-1911-3p was capable of regulating *MEAK7* mRNA levels, we sought to confirm the extent to which miR-1911-3p directly targeted the 3′ untranslated region (3′-UTR) of *MEAK7* mRNA. The target relationship between *MEAK7* mRNA and miR-1911-3p was investigated using a Dual Luciferase Assay. The 3′-UTR sequences of *MEAK7* mRNA were cloned into a pmirGLO dual-luciferase miRNA target expression vector and the empty pmirGLO vector (pmirGLO Control) was set to 100% for luciferase activity. There was no change in luciferase activity when H1975 or H1299 cells were co-transfected with pmiRGLO-3′-UTR *MEAK7* and miRNA controls (Figure 4, A and B). However, when H1975 or H1299 cells were co-transfected with pmiRGLO-3′-UTR *MEAK7* and miR-1911-3p mimic, luciferase activity was decreased by about 50% (Figure 4, A and B). In the presence of miR-1911-3p inhibitor and pmiRGLO-3′UTR mEAK-7, luciferase activity was not significantly increased (Figure 4, C and D) because both H1975 and H1299 cells have low levels of miR-1911-3p (Figure 1C). These results demonstrate that miR-1911-3p negatively regulated *MEAK7* mRNA by binding to its 3′-UTR (Figure 4E).

### 3.3 miR-1911-3p impairs co-localization of mTOR to the lysosome

**Figure 5:**
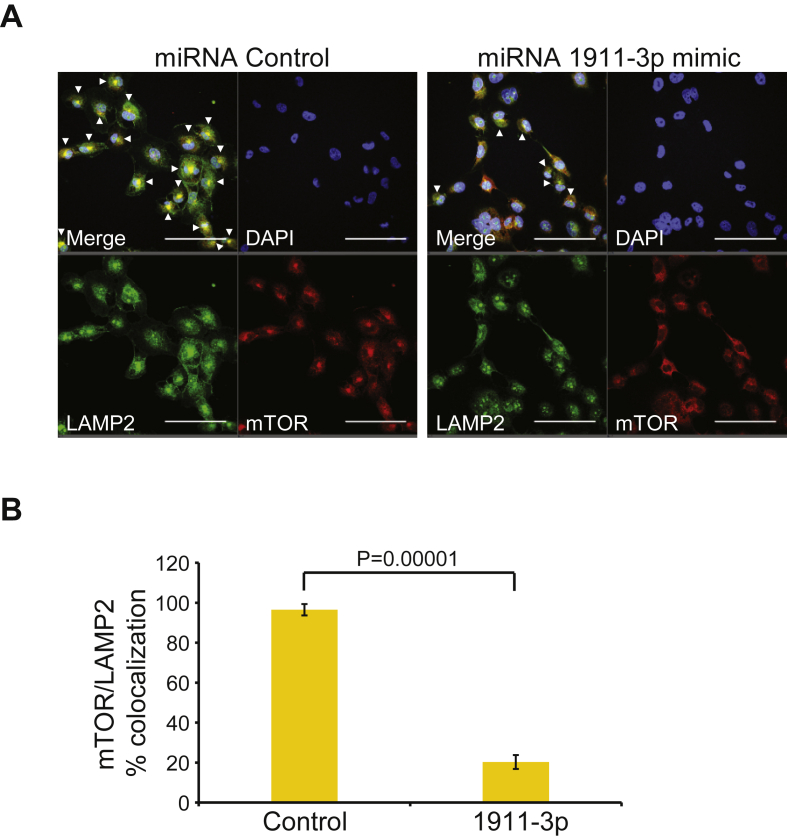
miR-1911-3p impairs co-localization of mTOR to the lysosome. (A) H1299 cells were treated with miRNA control or miR-1911-3p for 48 h. 250,000 cells were seeded onto glass well slides and allow to settle for 24 h. Cells were then starved for 1 h, refed with 10% FBS for 1 h, and processed for immunofluorescence analysis against mTOR and LAMP2 (lysosomal marker). White arrowheads indicate colocalization of mTOR and LAMP2. Scale bars, 100 μm. (B) Quantitative analysis of colocalization of mTOR and LAMP2 for (A). Six confocal images per condition were used for quantification.

We have previously found that mEAK-7 knockdown impaired mTOR localization to the lysosome [1], demonstrating that mEAK-7 is important for mTOR localization. Because miR-1911-3p negatively regulated *MEAK7* mRNA and protein levels, we hypothesized that miR-1911-3p may also influence mTOR localization to the lysosome. H1299 cells were transfected with miR-control or miR-1911-3p mimic for 72 h miR-1911-3p significantly decreased mTOR/LAMP2 (lysosomal marker) colocalization (Control:miR-1911-3p, fold change: 4.75) (Figure 5, A and B), confirming that miR-1911-3p is also important for mTOR localization to the lysosome through mEAK-7.

### 3.4 miR-1911-3p suppresses cell proliferation and migration in NSCLC cells

**Figure 6:**
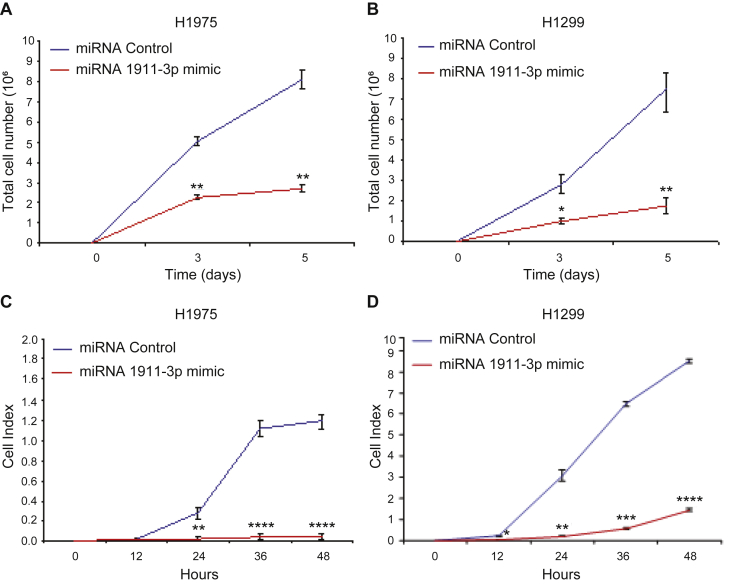
miR-1911-3p significantly decreases cell proliferation and migration in NSCLC cell lines. **(**A and B) cell proliferation: H1975 (A) and H1299 (B) cells were transfected with 100 nM of miR-control or miR-1911-3p mimic. 48 hours after transfection, 200,000 cells were seeded into 100 mm tissue culture dishes and counted after days 3 and 5 using the LUNA cell counter. (C and D) cell migration: H1975 (C) and H1299 (D) cells were transfected with 100 nM of miR-control or miR-1911-3p mimic. 48 hours after transfection, 50,000 cells were transferred to CIM 16-well plates and xCELLigence System was used to capture real-time cell motility for 48 h. Statistical significance: ∗P < 0.05, ∗∗P < 0.001, ∗∗∗P < 0.00001, ∗∗∗∗P < 0.000001.

**Figure 7:**
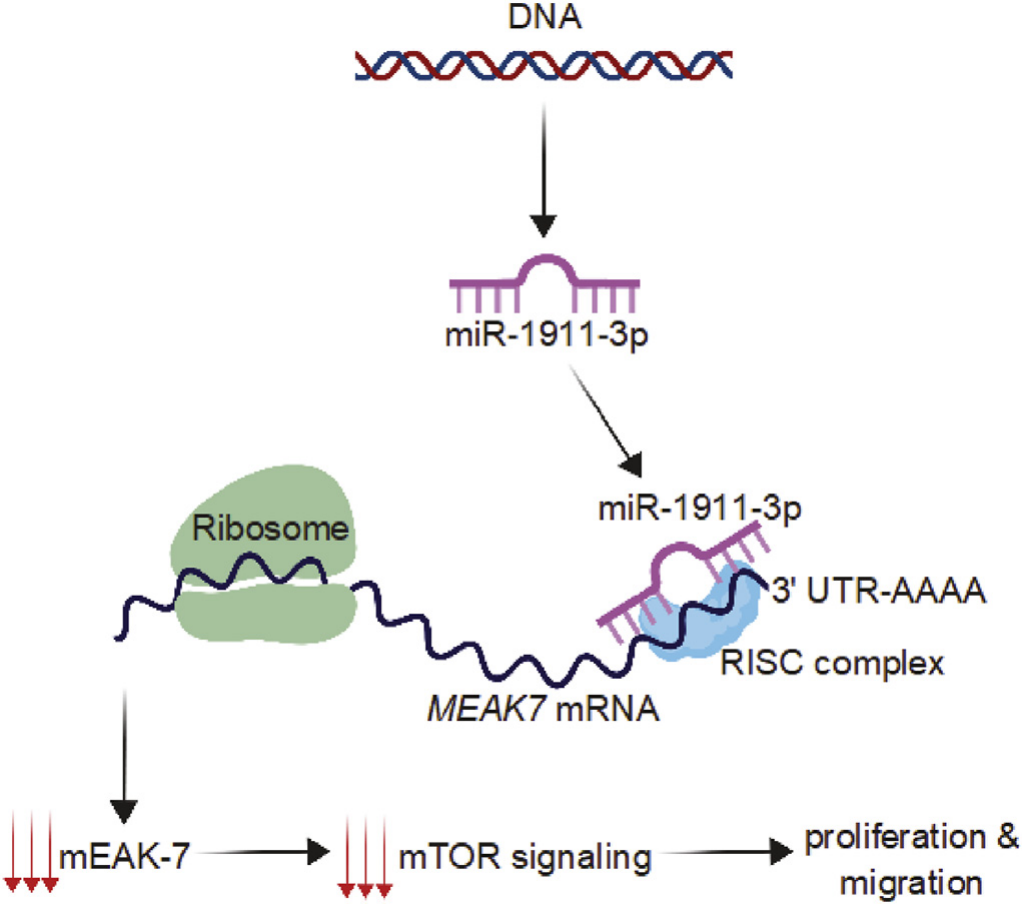
Model for miR-1911-3p function on mEAK-7-mediated mTOR signaling. miR-1911-3p is synthesized and binds to the 3′-UTR of *MEAK7* through the RISC complex. This binding down-regulates *MEAK7* translation and subsequently suppresses mTOR signaling in cells, resulting in a substantial decrease in cell proliferation and migration.

mTOR signaling is well known for its ability to regulate cell proliferation [25] and cell migration [26]. Likewise, mEAK-7 has been shown to be an essential effector of cell proliferation and migration in human cancer cells [1]. Because miR-1911-3p negatively regulated mEAK-7 expression and subsequent mTOR signaling, lung cancer cell proliferation and migration were analyzed in response to miR-1911-3p. H1975 and H1299 cells were transfected with miR-control or miR-1911-3p mimic for 48 h miR-1911-3p significantly decreased cell proliferation (Figure 6, A and B) and migration (Figure 6, C and D) in both H1975 and H1299 cells. Thus, miR-1911-3p suppressed mTOR signaling through down-regulation of mEAK-7, resulting in reduced cell proliferation and migration (Figure 7).

## 4 Discussion

Recent studies suggest multiple functions of miRNAs in NSCLC. For example, miR-99b acts as a tumor suppressor in NSCLC by directly targeting fibroblast growth factor receptor 3 [27]. Another study reported that miR-320a-3p regulates cell metastasis and invasion as a tumor suppressor through PI3K/Akt inactivation in NSCLC [28], and bioinformatics and functional analyses have been used to identify potential miRNAs and their regulatory mechanism in NSCLC [29] showing that some target genes of downregulated miRNAs are associated with PI3K-Akt signaling pathway, among other pathways. VEGFA and MYC were regulated by downregulated and upregulated miRNAs. In this study, we observed that miR-1911-3p caused a down-regulation of VEGF and MYC, which were involved with PI3K-Akt and mTOR signaling pathway (Figure 3, C and D). Besides PI3K-Akt and mTOR regulation, miRNAs influence Transforming Growth Factor-β1 (TGF-β1) signaling at multiple levels and the dysregulation of TGF-β1 signaling is often linked to several diseases, including cancer [30]. We found that miR-1911-3p caused an up-regulation of TGF-β1 in NSCLC (Figure 3B).

Because mTOR is a validated therapeutic target for cancer, miRNAs inhibiting mTOR signaling may provide a novel approach to facilitate an integrated anti-cancer therapy [22]. For example, miR-125b-5p is a potential therapeutic target of cancers with hyperactivated mTORC1 [31]. We recently identified a novel positive regulator of mTOR signaling, which we named mEAK-7 [1]. Here, we demonstrated that miR-1911-3p directly targets the 3′-UTR of *MEAK7* mRNA and negatively regulates *MEAK7* mRNA and mEAK-7 expression. Therefore, miRNA 1911-3p is able to significantly reduce mEAK-7-mediated mTORC1 signaling and suppress cell proliferation and migration in NSCLC cells, indicating a role for this miRNA as a tumor suppressor gene. To our knowledge, this is the first report linking miR-1911-3p to NSCLC and more importantly, linking miR-1911-3p to suppressing mTOR signaling through *MEAK7*. Thus it will be important to determine if manipulation of miR-1911-3p expression could have a potential therapeutic application in lung cancer.

## Declarations

### Author contribution statement

Paul H. Krebsbach: Conceived and designed the experiments; Analyzed and interpreted the data; Wrote the paper.

Daniela Baccelli Mendonça: Conceived and designed the experiments; Performed the experiments; Analyzed and interpreted the data; Wrote the paper.

Joe Truong Nguyen: Performed the experiments; Analyzed and interpreted the data; Wrote the paper.

Fatima Haidar, Alexandra Lucienne Fox, Connor Ray, Halimah Amatullah, Jin Koo Kim: Performed the experiments; Analyzed and interpreted the data.

Fei Liu: Analyzed and interpreted the data.

### Funding statement

This work was supported by the National Institute of Dental and Craniofacial Research (1F30DE026048-01, R01-DE016530, and T32-DE007057); the Stuart & Barbara Padnos Research Award from the Comprehensive Cancer Center at the University of Michigan.

### Data availability statement

Data included in supplementary material.

### Declaration of interests statement

The authors declare no conflict of interest.

### Additional information

No additional information is available for this paper.

## Appendix A Supplementary data

Final Supplementary Material.pdf **Figure S1**. miR-1911-3p is an effector for mEAK-7 and mTOR signaling in human lung cancer cells. H1975 (A) and H1299 (B) cells were transfected with miR-control or miR-1911-3p mimic for 48 h to evaluate mEAK-7 and mTOR signaling. GAPDH was used as a loading control. **Figure S2**. miR-1911-3p is an effector for mEAK-7 and mTOR signaling in human lung cancer cells. Expression of mEAK-7 and mTOR signaling in H1299, H1975, and BEAS-2B cells was analyzed by Western blotting. β-actin was used as a loading control. Final Supplementary Material.pdf
