# Supplementary material for: MicroRNA-1911-3p targets mEAK-7 to suppress mTOR signaling in human lung cancer cells
Source: Heliyon. 2020 Dec 19;6(12):e05734. doi: 10.1016/j.heliyon.2020.e05734 (PMC7753913; doi:10.1016/j.heliyon.2020.e05734)
Supplement: Final Supplementary Material.pdf — Figure S1. miR-1911-3p is an effector for mEAK-7 and mTOR signaling in human lung cancer cells. H1975 (A) and H1299 (B) cells were transfected with miR-control or miR-1911-3p mimic for 48 h to evaluate mEAK-7 and mTOR signaling. GAPDH was used as a loading control. Figure S2. miR-1911-3p is an effector for mEAK-7 and mTOR signaling in human lung cancer cells. Expression of mEAK-7 and mTOR signaling in H1299, H1975, and BEAS-2B cells was analyzed by Western blotting. β-actin was used as a loading control. [file mmc1.pdf]

## Figure S1

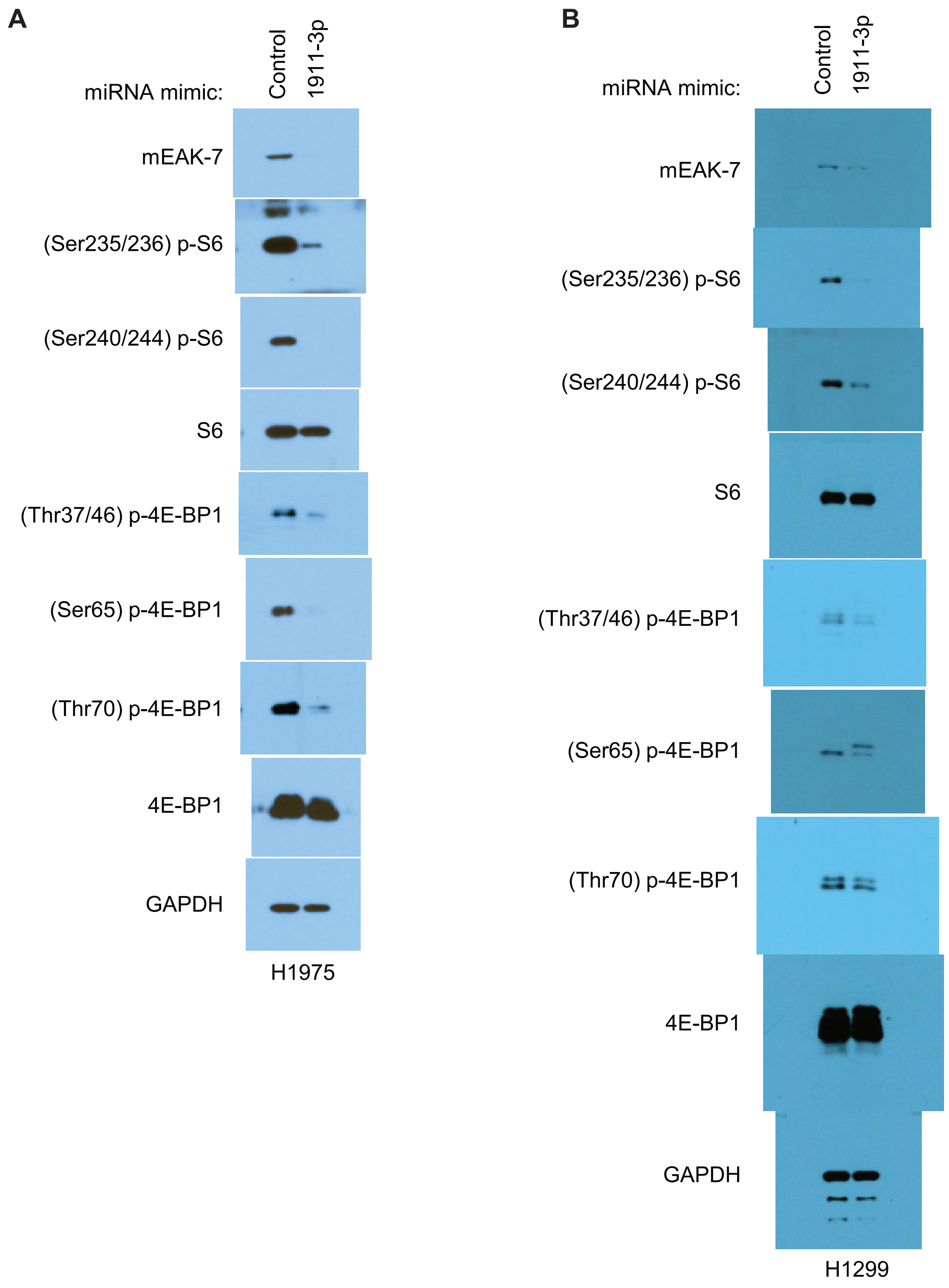


**Figure S1.** miR-1911-3p is an effector for mEAK-7 and mTOR signaling in human lung cancer cells. H1975 (A) and H1299 (B) cells were transfected with miR-1911-3p mimic for 48 hours to evaluate mEAK-7 and mTOR signaling. GAPDH was used as a loading control.

**Figure S2**

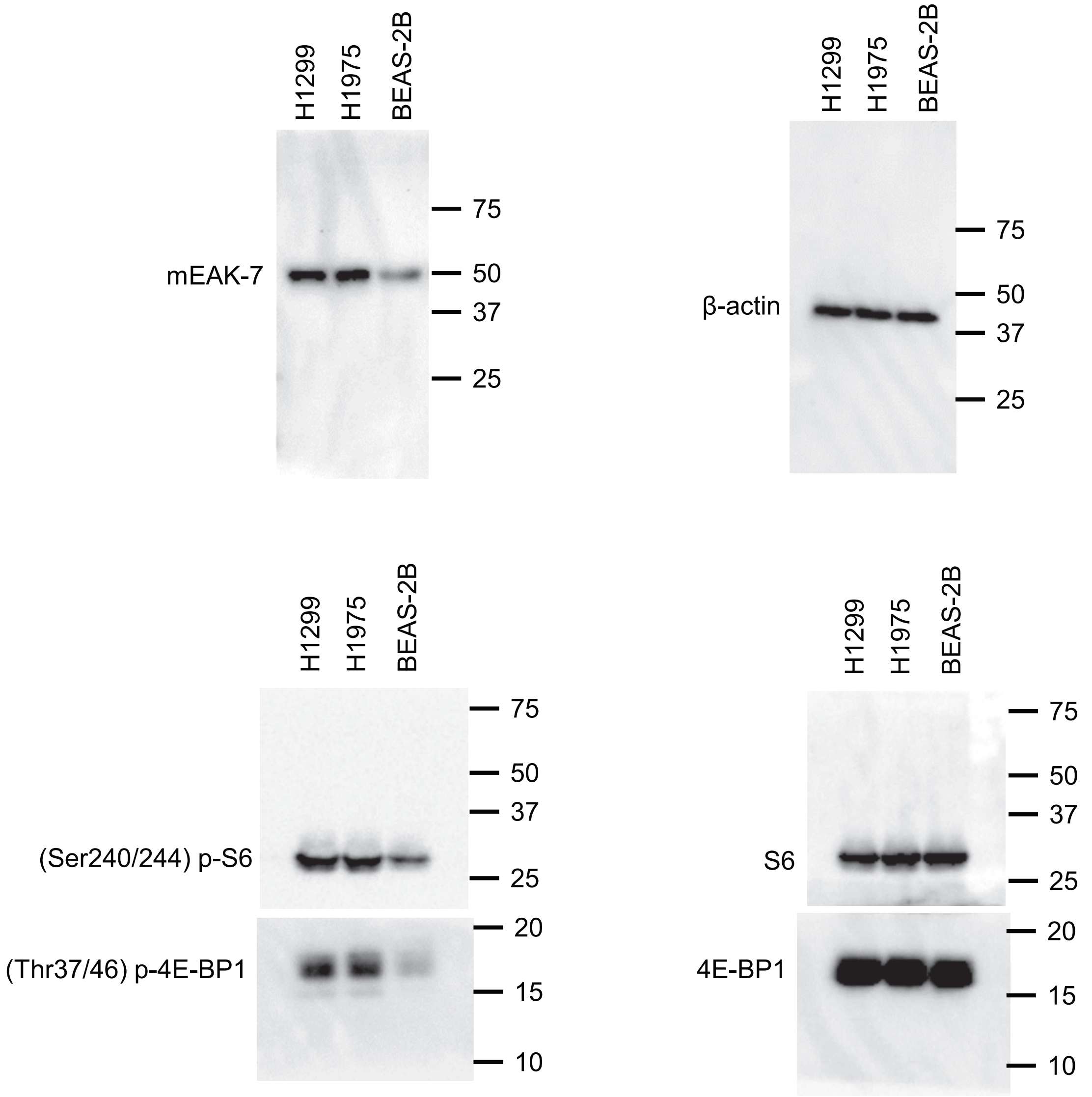


**Figure S2.** miR-1911-3p is an effector for mEAK-7 and mTOR signaling in human lung cancer cells. Expression of mEAK-7 and mTOR signaling in H1299, H1975, and BEAS-2B cells was analyzed by Western blotting. β-actin was used as a loading control.
